# Association of muscle mass measured by D_3_-Creatine (D_3_Cr), sarcopenic obesity, and insulin-glucose homeostasis in postmenopausal women

**DOI:** 10.1371/journal.pone.0278723

**Published:** 2022-12-09

**Authors:** Hailey R. Banack, Michael J. LaMonte, JoAnn E. Manson, Kexin Zhu, William J. Evans, Mahalakshmi Shankaran, Jean Wactawski-Wende

**Affiliations:** 1 Epidemiology Division, Dalla Lana School of Public Health, University of Toronto, Toronto, Ontario, Canada; 2 School of Public Health and Health Professions, University at Buffalo, Buffalo, New York, United States of America; 3 Division of Preventive Medicine, Department of Medicine, Brigham and Women’s Hospital, Harvard Medical School, Boston, Massachusetts, United States of America; 4 Department of Epidemiology, Harvard T.H. Chan School of Public Health, Boston, Massachusetts, United States of America; 5 Duke University Medical Center, Division of Geriatrics, Durham, North Carolina, United States of America; 6 Department of Nutritional Sciences and Toxicology, University of California, Berkeley, California, United States of America; University of Mississippi, UNITED STATES

## Abstract

The D_3_-Creatine (D_3_Cr) dilution method is a direct and accurate measure of skeletal muscle mass. In this study, we examined the association of D_3_Cr muscle mass with measures of insulin-glucose homeostasis in community dwelling postmenopausal women. Additionally, we examined association of sarcopenic obesity, defined as low D_3_Cr muscle mass and high percent body fat, with fasting plasma glucose, insulin, hemoglobin A1c and insulin resistance. Insulin resistance was measured by the homeostatic measure of insulin resistance (HOMA-IR). This pilot study included 74 participants (mean age = 82.3 years) from the Women’s Health Initiative-Buffalo site. The D_3_Cr method was initiated at a clinic visit and used to measure muscle mass via remote urine sample collection. Descriptive and graphical approaches and age-adjusted linear regression models were used to analyze study data. We examined muscle mass as an absolute value (kg) and scaled to body weight (D_3_Cr muscle mass/kg). There was an inverse relationship between skeletal muscle mass, and impaired insulin-glucose homeostasis. Women with low muscle mass had higher levels of insulin (uIU/mL; β = -0.40; 95% CI: -0.79, -0.01), fasting plasma glucose (mg/dL; β = -0.1; 95% CI: -0.2, 0.03), HbA1c (%; β = -2.30; 95% CI: -5.7, 1.1), and calculated homeostatic model of insulin resistance, HOMA-IR, (β = -1.49; 95% CI: -2.9, -0.1). Sarcopenic obesity was common in this population of women; 41% of participants were categorized as having low muscle mass and high percent body fat. Results demonstrate that D_3_Cr muscle mass is independently associated with measures of insulin-glucose homeostasis, but obesity is a stronger predictor of insulin resistance than muscle mass.

## Introduction

Type 2 diabetes (T2D) is highly prevalent in older adults [1]. More than one in five older adults in the United States have diagnosed T2D and a further 30% have impaired glucose tolerance [1]. Historically, men have had higher rates of T2D than women, however recent data show: a) increasing incidence of T2D in women, closing the gender gap in diabetes prevalence and b) projected rates of T2D incidence will be higher for women than men [2].

Sarcopenia (low muscle mass) and obesity are also very common in older adults. Obesity is estimated to affect more than 41% of adults over age 60 [3]. Estimates of the prevalence of sarcopenia vary by the definition used, but recent estimates indicate it affects between 25% and 45% of older adults [4–6]. Sarcopenia is an important health risk for older women in particular. After menopause, women lose 1–3% of their lean body mass per year [6]. Prior research has demonstrated that sarcopenia and obesity may act synergistically to increase the risk of negative health consequences of either condition independently [7]. This phenomenon, termed sarcopenic obesity, has been documented to increase the risk of diabetes and cardiovascular disease [8].

Most prior research examining sarcopenia and sarcopenic obesity has used lean body mass, often from DXA scans, as a surrogate measure of skeletal muscle mass [9]. However, lean body mass and skeletal muscle mass are not equivalent; skeletal muscle mass is only one component of lean body mass [9]. Lean mass from DXA includes fibrotic, vascular, and connective tissues and organs, and also depends on hydration status [9]. DXA also estimates lean mass by subtraction: it directly estimates fat mass and bone mineral content, but calculates lean body mass as total mass minus bone and fat mass [10]. Skeletal muscle mass can be measured directly via the D_3_-creatine (D_3_Cr) dilution method [10–12]. The D_3_Cr dilution method is a non-invasive, direct measure of muscle mass that can be used remotely (at home) and has been validated in laboratory and clinical settings [13, 14]. We previously reported a moderate positive correlation between D_3_Cr muscle mass DXA lean body mass (*r* = 0.50) and appendicular lean mass (*r* = 0.50) from DXA in older women [15]. These results support previous findings that DXA measures of lean body mass and skeletal muscle mass from D_3_Cr are not equivalent [9, 15].

Orwoll and colleagues recently examined the association between sarcopenic obesity, including percent body fat measured by DXA and skeletal muscle mass measured via D_3_Cr, and physical performance in a sample of older men [9]. Their results demonstrated that D_3_Cr muscle mass was strongly associated with functional limitation, physical performance, and falls, but fat mass (obesity) had minimal influence on these outcomes after muscle mass was accounted for [9]. These results highlight the relative importance of muscle mass compared to fat mass among individuals with sarcopenic obesity when considering functional outcomes. However, the relationship between sarcopenia, measured by D_3_Cr muscle mass, and D_3_Cr sarcopenic obesity, with insulin-glucose homeostasis, has not been reported. Moreover, findings describing D_3_Cr sarcopenic obesity have been restricted to studies in men.

### Pathophysiologic link between muscle mass and diabetes

There is a complex, bidirectional relationship between muscle mass and diabetes status. Impaired insulin-glucose homeostasis may contribute to loss of muscle mass, while loss of muscle mass may result in worsening dysglycemia, insulin resistance, and T2D. Skeletal muscle is the primary site of insulin-mediated glucose disposal as approximately 80% of glucose uptake occurs in muscle tissue [16, 17]. As blood glucose increases, insulin lowers circulating glucose [18]. Insulin promotes glycogenesis (glucose storage) and uptake of glucose into muscle [19]. As such, skeletal muscle plays a critical role in maintaining insulin-glucose homeostasis. Chronic disruption of insulin-glucose homeostasis results in hyperglycemia, hyperinsulinemia, and insulin resistance; these pathophysiologic abnormalities precede the onset of diabetes. Insulin resistance occurs when cells do not respond properly to insulin secreted by the pancreas, insulin no longer stimulates glucose uptake and utilization in muscle tissue, resulting in excess glucose in the blood [20]. Insulin-stimulated muscle glucose uptake is substantially (>50%) reduced among individuals with impaired glucose tolerance and T2D [16] This supports an important hypothesis that a defect in muscle insulin action is found in those with T2D. Skeletal muscle insulin resistance is considered to be the primary defect in individuals with T2D, and is often evident long before overt hyperglycemia or T2D develop [16].

Given the high burden of sarcopenia and diabetes in older adults, a better understanding of putative mechanistic pathways is a step toward the development of intervention programs. Moreover, the extent to which sarcopenic obesity increases the risk of dysglycemia, and insulin resistance, compared to sarcopenia or obesity alone has not been described. Here we present the results of a pilot study examining the cross-sectional association between skeletal muscle mass measured by D_3_Cr, sarcopenic obesity, and insulin-glucose homeostasis in a sample of community-dwelling postmenopausal women from the Buffalo site of the Women’s Health Initiative (WHI).

## Methods

We recruited a sample of community dwelling postmenopausal women (n = 74) from the WHI Buffalo, New York clinical site. Details of the WHI study have been described previously [21, 22]. For this pilot study, all WHI participants living within 50 miles of the WHI-Buffalo site were eligible; a simple random sample was used to select women to participate in this study. There were no additional exclusion criteria.

Participants were invited to join the study via mailed recruitment package which included a cover letter explaining the study, an informed consent form, and questionnaire packet. Women were subsequently contacted by telephone and those who agreed to participate were invited to a clinic visit at the WHI Buffalo study site. The clinic visit followed a standard protocol and was approximately one hour in length. Clinic visits consisted of consent, a fasting blood draw, anthropometric measures, including height and weight, blood pressure measurement, and a whole body DXA scan (Hologic QDR-4500). The blood sample was drawn by venipuncture by a trained phlebotomist. Vials were processed immediately and frozen onsite. Samples were analyzed by an external laboratory (Kaleida Health, Buffalo, NY) and tests included a comprehensive metabolic panel, hemoglobin A1c (HbA1c), insulin, lipid panel, and complete blood count. Anthropometric measures (height, weight, hip and waist circumference) were obtained at the clinic visit by trained WHI clinic staff using standardized protocols. BMI was calculated as weight divided by height squared (kg/m^2^). Systolic and diastolic blood pressure were measured after 5 min of quiet sitting with legs uncrossed using a manual sphygmomanometer, Hypertension was defined as a systolic pressure ≥140 mm Hg and/or a diastolic pressure ≥90 mm Hg.

This study was approved by the IRB at the University at Buffalo; all participants provided written informed consent.

### D_3_Cr dilution method

Muscle mass was measured with the D_3_ Cr dilution method. Dilution of a stable isotope-labeled creatine, and measurement of urine creatinine enrichment, provides an accurate direct measure of muscle mass [10]. The D_3_-procedure involves several steps [1]. Participants were given a single oral dose of deuterated creatine (D_3_Cr). D_3_Cr is transported into skeletal muscle where it is turned over via isomerization to D_3_-creatinine and rapidly excreted. Creatine is synthesized in the liver and kidneys and transported into the sarcomere [37]. Total body creatine is proportional to skeletal muscle mass [37]. Creatine is commonly consumed via diet or supplements (approximately 1000 mg/d of creatine in regular diet from animal protein sources) [38]. D_3_-creatine is similar to naturally occurring creatine, but the hydrogen atoms have been replaced by deuterium, a stable, non-radioactive isotope of hydrogen with one proton and one neutron in its nucleus. Replacing the hydrogen atoms with deuterium allows it to be ‘labeled’ for comparison with ‘unlabeled’ natural creatine. Creatinine (or D_3_-creatinine) diffuses from skeletal muscle and is excreted in urine, where it can be measured using high performance liquid chromatography and mass spectrometry [31]. The ratio of labeled D_3_-creatinine to unlabeled creatinine in urine provides a measure of creatine pool size and of skeletal muscle mass [1]. Muscle contains approximately 4.3 gm/kg [1, 37]. A recent review of the D_3_Cr method highlighted key considerations related to the use of D_3_Cr method for measuring muscle mass related to D3Cr absorption, retention, distribution and skeletal muscle creatine concentration [23].

**Fig 1:**
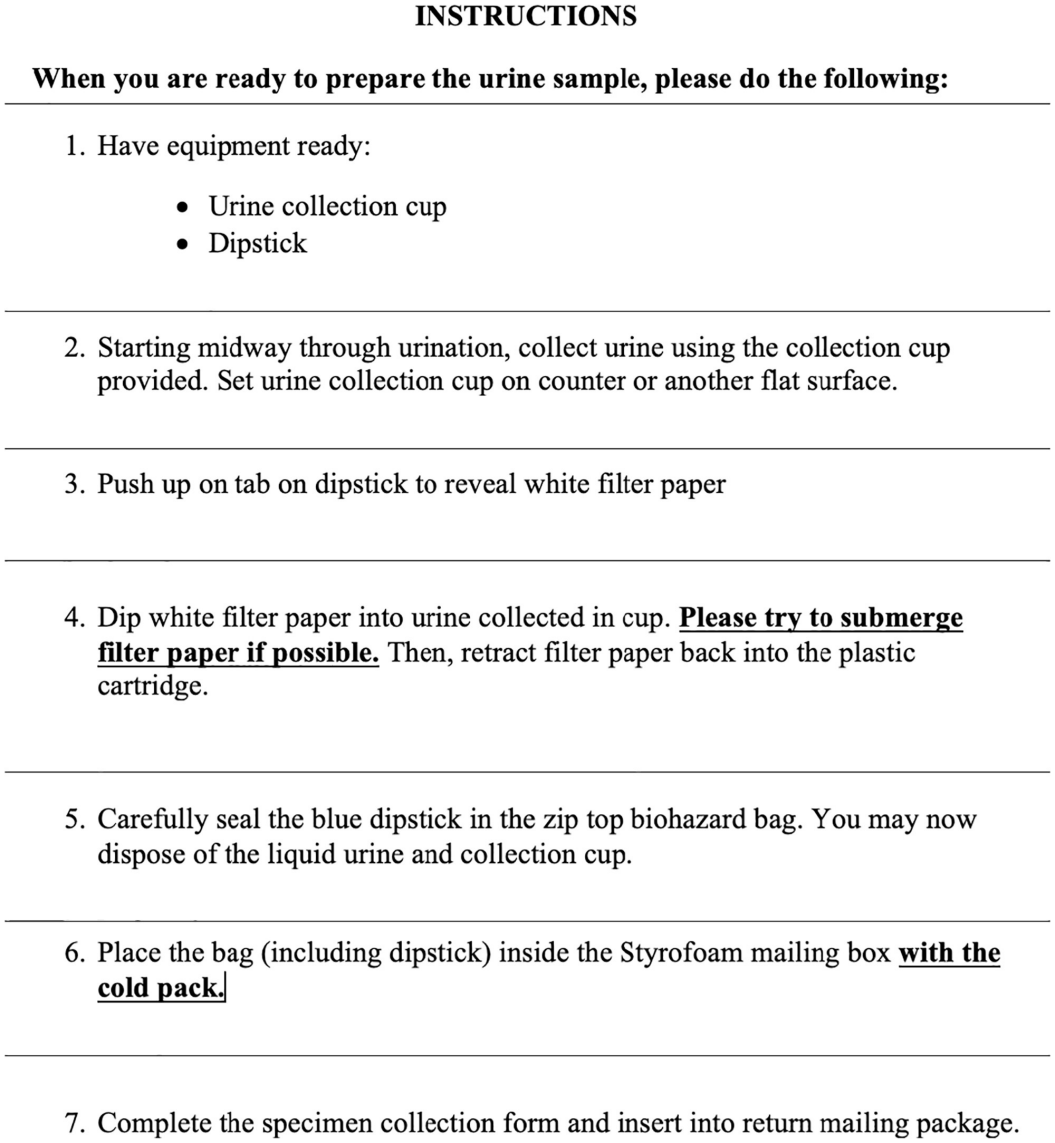
Urine sample collection instructions.

In this study, participants consumed an oral dose of 30 mg D_3_Cr (Cambridge Isotope Laboratories, Inc. Tewksbury, MA; encapsulated by Valor Compounding Pharmacy, Berkeley, CA). They were sent home with a urine sample collection kit, collection instructions, and return mailing instructions. The urine collection kit included a dipstick, urine collection cup, styrofoam return mailer, ice pack, and pre-filled FedEx mailing label. A research team member recorded the date and time the pill was consumed. Participants were asked to collect a fasting morning urine sample on a specific date between 72 and 144 hours after their clinic visit using the urine cup provided. As described in the instructions, participants extended the filter paper contained within the dipstick into the urine sample, fully submerging the paper, and then retracted the dipstick (see Fig 1). They were instructed to dispose of the urine and cup and place the dipstick into a zip-top biohazard bag. After sample collection, participants packed the dipstick with an ice pack into a Styrofoam mailer and called FedEx for a same-day at-home pick-up. Samples were sent via overnight shipping and arrived at the WHI Buffalo study center less than 24 hours after sample collection. The D_3_Cr protocol has been described previously [13–15]. Additional information, including copies of instructions provided to participants and reminder checklists are included in the Supplemental Appendix S1 in S1 File.

At a 1-year follow-up visit, a convenience sample of 21 repeat participants completed the D_3_Cr protocol entirely remotely, with no clinic visit. Study kits were mailed to participants with consent forms, the D_3_Cr pill, urine sample collection materials, ice pack and supplies to return the sample via FedEx. We provided detailed instructions, with pictures. We also called the participants twice, once to remind them to take the D_3_Cr capsule on a specific date as outlined in their study kit and the second time in the evening before the overnight fast for their morning urine sample.

Upon arrival at the University at Buffalo, all samples were visually inspected and deemed to be in good condition. Specimen were stored in -20°C freezers in a Biospecimen Bank. Samples were sent in batches on dry ice by overnight shipping to the University of California, Berkeley for analysis. Urinary creatine, creatinine, and D_3_-creatinine were measured by liquid chromatography-tandem mass spectrometry, and skeletal muscle mass was estimated by a validated algorithm [12].

## Measures

We examined D_3_Cr muscle mass as absolute (kg) and relative (D_3_Cr muscle mass/ kg body mass) measures. Absolute and relative muscle mass were examined as continuous variables and also categorized as low and high by median split. This dichotomous parameterization was used to enhance the comparability of our study results with prior research on D_3_Cr muscle mass [14]. Percent total body fat from DXA was used as a measure of obesity status. Women with greater than 35% body fat were deemed to be obese, while women with less than 35% body fat were considered non-obese [24]. We also considered WHO cut-points for obesity defined by BMI (BMI>30kg/m^2^) and waist circumference (women >88cm) [25]. Validated WHI questionnaires were used to measure physical activity levels. Participants were asked to self-report how often they engage in different types of physical activity, such as walking outside of the home for more than 10 minutes without stopping, strenuous or very strenuous exercise (e.g., aerobics, jogging, tennis, swimming laps), and moderate exercise (e.g., biking outdoors, calisthenics, easy swimming, dancing).

Insulin-glucose homeostasis was assessed via blood glucose, insulin, and hemoglobin A1c (HbA1c) from the fasting blood draw. We also calculated the homeostatic measure of insulin resistance (HOMA-IR) [26].

## Statistical analysis

Statistical analyses included examination of descriptive characteristics of the study population overall and by muscle mass category. To examine the association between skeletal muscle mass measured by D_3_Cr with measures of insulin-glucose homeostasis, we examined Pearson correlation coefficients and linear regression models.

In our primary analyses, to examine sarcopenic obesity, we cross-classified women according to their D_3_Cr muscle mass and percent body fat category, resulting in four groups of women: low muscle mass/non-obese, high muscle mass/non-obese, low muscle mass/obese, high muscle mass/obese. We additionally describe the prevalence of sarcopenic obesity in this population when BMI and waist circumference cut-points are used to define obesity. Given the sample size in this pilot study (n = 74), we were conservative with our analyses. We examined the relation of the categories representing sarcopenic obesity with indices of insulin/glucose homeostasis using descriptive approaches. This analysis is intended to provide preliminary support for the hypothesis that low muscle mass is associated with worse insulin-glucose homeostasis, independently of obesity status.

## Results

**Fig 2:**
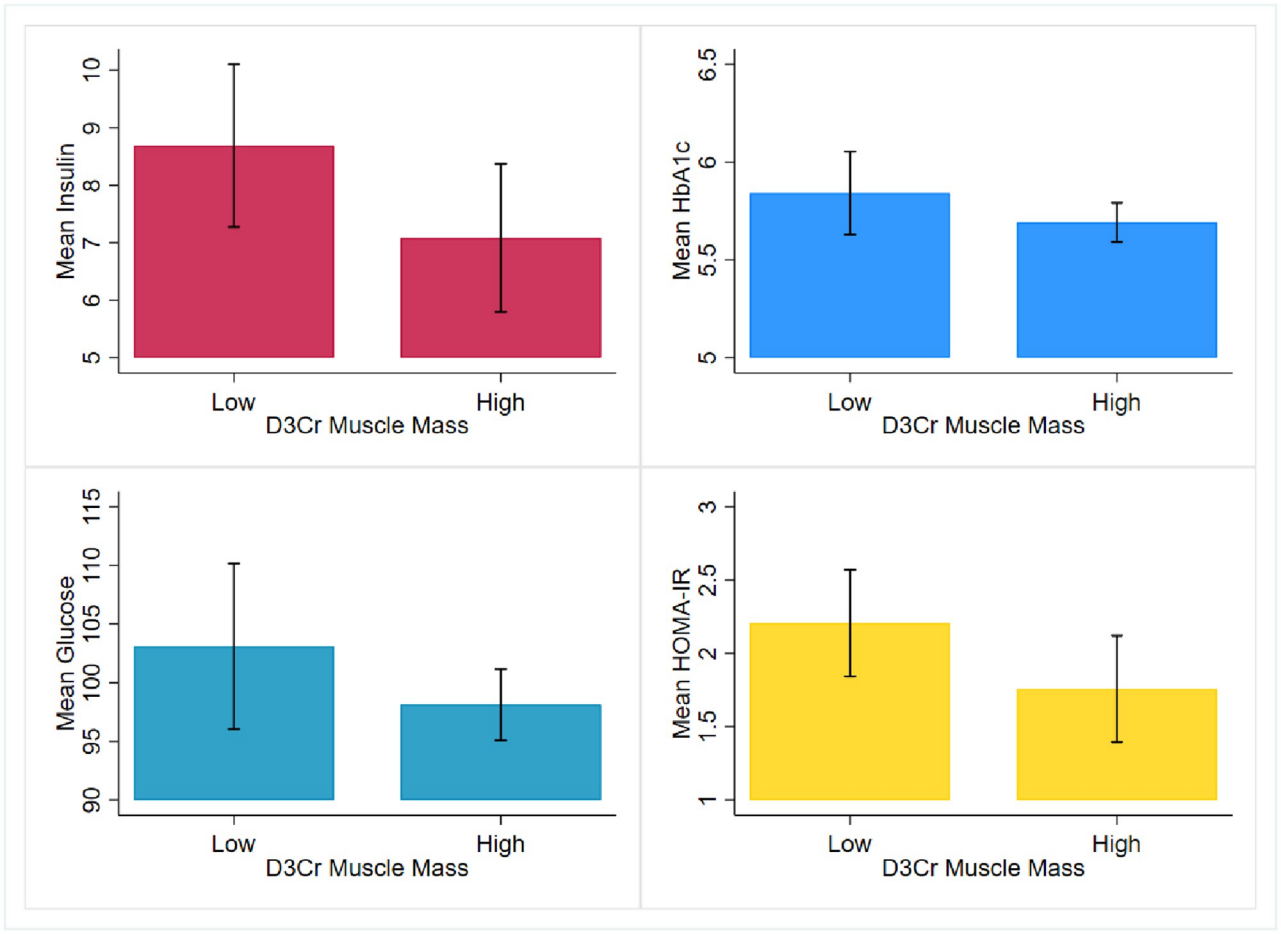
Comparison of mean values of insulin, HbA1c, glucose and HOMA-IR among postmenopausal women with low and high D_3_Cr muscle mass.

We received 73 out of 74 (99%) urine samples from study participants at baseline and 100% of samples at 1-year follow-up (all 21 samples were returned). Descriptive characteristics of the study sample are presented in Table 1 overall and stratified by low and high muscle mass categories. At baseline, the mean age of the study sample was 82 years (SD ± 5.4 years; range: 74 to 96) and 93% were non-Hispanic white (n = 68). Participants had a mean of 18.0kg ± 3.5 (range 12.3 to 30.1kg) muscle mass and 28.0% ± 5.9 (range 17.3 to 45.9%) relative muscle mass, scaled to body weight (D_3_Cr/kg). Results in Table 1 describe an inverse relationship between D_3_Cr muscle mass and glycemic control. Blood glucose measures were, on average, 103.1 (±18.6) in the low muscle mass group and 98.1 (±8.9) in the high muscle mass group. Corresponding insulin measures were 8.69 (±3.7) and 7.09 (±3.7) and HOMA-IR was 2.21 (±0.96) and 1.75 (±1.1) in the low and high muscle mass groups, respectively. Of the women who had an HbA1c less than 5.7, 17 women (68%) had high muscle mass while 8 (32%) had low muscle mass. Of those who had HbA1c greater than 5.7, more women had low muscle mass (n = 21) than high (n = 18). Mean HbA1c values are below the pre-diabetes cut point in the low muscle mass group (5.8 ± 0.6). Fig 2 provides a graphical demonstration of mean differences in insulin, HbA1c, glucose, and HOMA-IR across low and high muscle groups. Women in the low muscle mass group had higher values of blood glucose, HbA1c, insulin, and HOMA-IR.

**Table 1:** Descriptive characteristics of the study sample and indices of insulin-glucose homeostasis in the total sample and by D_3_Cr muscle mass categorized by median split mean(sd) or n(%).

	D_3_Cr muscle mass				Overall D_3_Cr muscle mass (n = 73)	
	Low (n = 36)		High (n = 37)			
**Mean D3Cr/Kg**	23.4	(2.65)	32.5	(4.49)	28.0	(5.90)
**Age**	82.78	(5.75)	81.92	(5.10)	82.34	(5.37)
**Ethnicity**						
**Non-white**	2	(40.00)	3	(60.00)	5	(100)
**White**	34	(50.00)	34	(50.00)	68	(100)
**BMI (kg/m** ^**2**^ **)**	27.68	(4.79)	24.4	(3.39)	25.95	(4.44)
**18.5 to <25**	12	(31.58)	26	(68.42)	38	(100)
**25 to < 30**	14	(66.67)	7	(33.33)	21	(100)
**≥ 30**	10	(71.43)	4	(28.57)	14	(100)
**Waist Circumference (cm)**	90.94	(13.77)	82.82	(10.39)	86.75	(12.69)
**Hip Circumference (cm)**	105.87	(9.34)	98.55	(6.08)	102.08	(8.60)
**Waist-to-hip ratio**	0.86	(0.10)	0.84	(0.08)	0.85	(0.09)
DXA Lean body mass (per kg), mean (SD)	0.59	(0.05)	0.64	(0.05)	0.61	(0.05)
DXA Appendicular lean mass (per kg), mean (SD)	0.25	(0.02)	0.27	(0.03)	0.26	(0.03)
DXA Percent body fat (%), mean (SD)	38.4	5.3	33.8	5.10	36.1	5.6
**SBP (mmHg)**	135.03	(15.88)	139.86	(18.19)	137.73	(17.16)
**DBP (mmHg)**	72.78	(7.84)	75.43	(8.97)	74.2	(8.45)
**Glucose (mg/dL)**	103.1	(18.57)	98.11	(8.86)	100.35	(14.10)
**Hemoglobin A1c (%)**	5.84	(0.56)	5.69	(0.29)	5.76	(0.43)
**< 5.7**	8	(32.00)	17	(68.00)	25	(100)
**≥ 5.7**	21	(53.85)	18	(46.15)	39	(100)
**Insulin Level (uIU/mL)**	8.69	(3.72)	7.09	(3.74)	7.77	(3.78)
**HOMA-IR**	2.21	(0.96)	1.75	(1.05)	1.95	(1.02)
**TG-HDL ratio**	2.0	(1.24)	1.87	(1.94)	1.91	(1.64)
**Physical activity (MET-hours/wk)**	12.7	(12.2)	18.7	(13.2)	16.6	(14.8)

Women with high D_3_Cr muscle mass reported higher overall levels of physical activity measured by MET-hours per week than women with low muscle mass. Additionally, women with low D_3_Cr muscle mass were more likely to report doing no walking, moderate or strenuous exercise than women with high muscle mass (Table 2).

**Table 2:** Comparison of self-report exercise and physical activity patterns among women with high and low muscle mass.

	D_3_Cr muscle mass				
	Low (n = 36)		High (n = 37)		P-value
Frequency of physical activity	n	Percent (%)	n	Percent (%)	
**Walking**					0.19
None	10	27.8	6	16.2	
1–2 days per week	14	38.9	12	32.4	
>3 days per week	10	27.8	15	40.3	
**Moderate exercise**					0.88
None	18	50.0	14	37.8	
1–2 days per week	7	19.4	13	35.1	
>3 days per week	11	30.6	10	27.1	
**Strenuous exercise**					0.84
None	24	66.7	22	59.5	
1–2 days per week	6	16.7	9	24.3	
>3 days per week	6	16.7	6	16.2	

**Fig 3:**
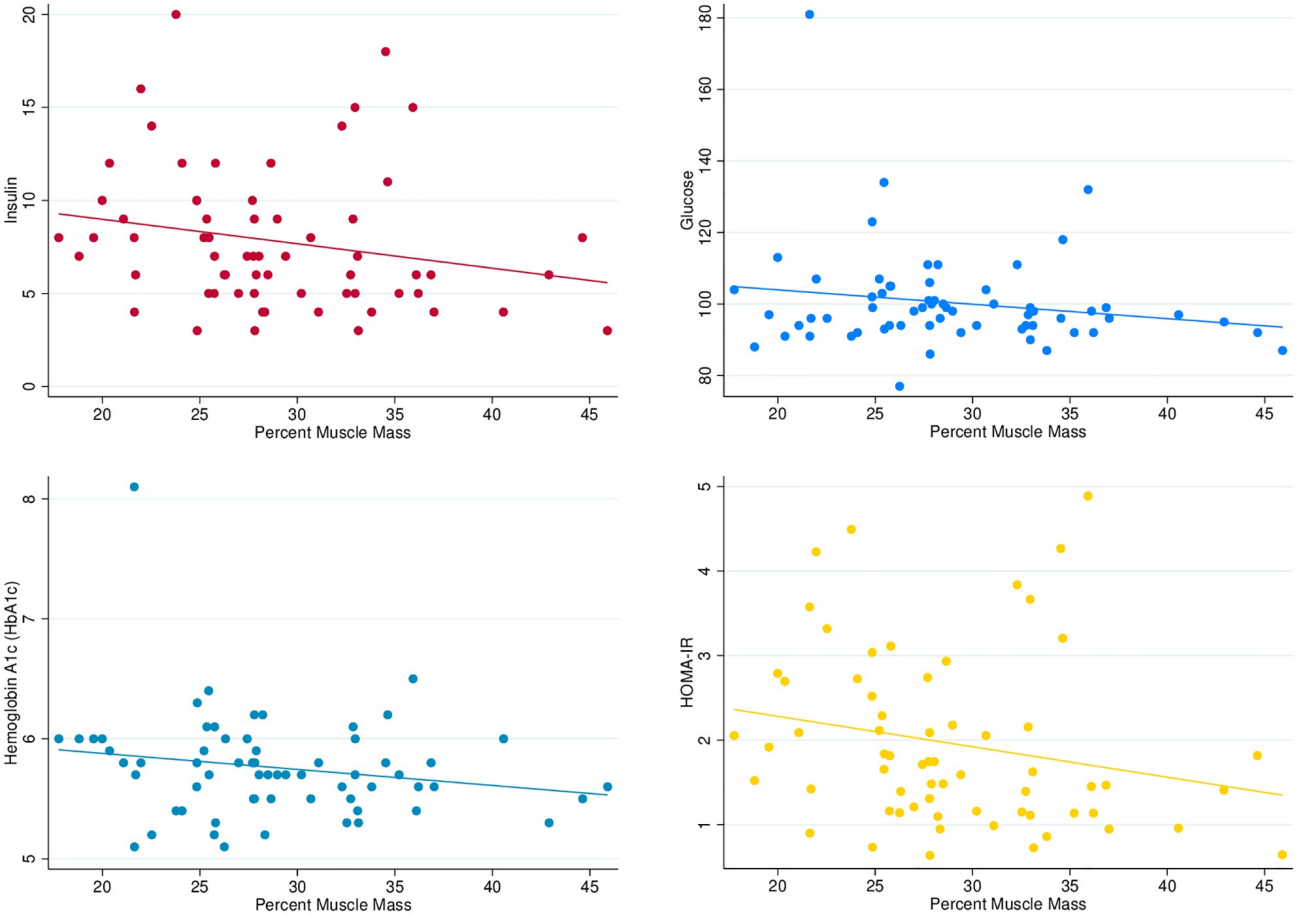
Scatterplots describing the association between indices of insulin-glucose homeostasis and percent muscle mass.

Pairwise correlation coefficients for relative (%) D_3_Cr muscle mass were -0.18 for glucose (p = 0.15), -0.22 for insulin (p = 0.07), -0.18 for HbA1c (p = 0.16) and -0.23 for HOMA-IR (p = 0.07). See Table 3 for the full correlation matrix describing association between these variables. In addition, Fig 3 describes the relationship between indices of insulin-glucose homeostasis and percent D_3_Cr muscle mass including a linear prediction line and 95% confidence interval. This provides graphical support for the inverse relationship between muscle mass and insulin-glucose homeostasis across categories of muscle mass above. Results from crude and adjusted linear regression models are presented in Table 4. Age-adjusted linear regression models suggest an association between insulin-glucose homeostasis and D_3_Cr muscle mass: insulin (β = -0.40; 95% CI: -0.79, -0.01), HbA1c (β = -2.30; 95% CI: -5.7, 1.1), glucose (β = -0.1; 95% CI: -0.2, 0.03), and HOMA-IR (β = -1.49; 95% CI: -2.9, -0.1).

**Table 3:** Correlation matrix for D3Cr muscle mass and indices of insulin-glucose homeostasis.

	**D**_**3**_ **Cr muscle mass**	**Glucose**	**Insulin**	**HbA1c**	**HOMA-IR**
**D**_**3**_ **Cr muscle mass**	**1.00**				
**Glucose**	**-0.18**	**1.00**			
**Insulin**	**-0.23**	**0.19**	**1.00**		
**HbA1c**	**-0.18**	**0.77**	**0.01**	**1.00**	
**HOMA-IR**	**-0.23**	**0.45**	**0.96**	**0.22**	**1.00**

**Table 4:** Beta coefficients and 95% confidence intervals from linear regression models describing the relationship between insulin-glucose homeostasis and D3Cr muscle mass.

	Model 1: Crude Model	Model 2: Age-Adjusted Model
Insulin	-0.35 (-0.73, 0.03)	-0.40 (-0.80, 0.01)
Glucose	-0.07 (-0.18, 0.03)	-0.08 (-5.7, 1.1)
HbA1c	-2.44 (-5.8, 0.95)	-2.30 (-0.18, 0.03)
HOMA-IR	-1.32 (-2.7, 0.10)	-1.49 (-2.93, -0.05)

There was a negative correlation between D_3_Cr muscle mass and percent body fat (r = -0.62), BMI (r = -0.52), and waist circumference (r = -0.45). There were 30 women with sarcopenic obesity (low D_3_Cr muscle mass, obese) defined according to total body fat >35% from DXA. There were 10 women with sarcopenic obesity when a BMI cut point (BMI>30kg/m2) was used to define the obesity component, and 22 women when waist circumference >88cm was used.

Results describing the cross tabulation of sarcopenia and obesity are presented in Table 5. Using DXA percent body fat to define obesity, women with sarcopenic obesity represented the largest group within the sample (41%), compared to 8% low muscle mass/non-obese, 32% high muscle mass/non-obese, and 19% high muscle mass/obese. Women with obesity had markedly higher values of insulin and HOMA-IR than women without obesity, regardless of muscle mass. Among non-obese women, having high muscle mass resulted in lower blood glucose levels (95 ± 5.4) compared to women with low muscle mass (106.8 ± 18.9), and lower insulin, HbA1c and HOMA-IR values. Additional results describing the association of sarcopenic obesity using WHO thresholds for obesity (using BMI and waist circumference) with indices of insulin-glucose homeostasis are included in the Supplemental Appendix S2 in S1 File.

**Table 5:** Comparison of indices of insulin-glucose homeostasis (mean ± SD) and sarcopenic obesity defined by D_3_Cr muscle mass and percent body fat.

Sarcopenic obesity [with obesity defined by DXA whole body fat (>35%)]				
	Low muscle mass / non-obese (n = 6)	Low muscle mass / obese (n = 30)	High muscle mass / non-obese (n = 23)	High muscle mass / obese (n = 14)
Blood glucose	106.8 (18.9)	102.5 (18.8)	95 (5.4)	104.1 (11.2)
Insulin	5.8 (0.96)	9.2 (3.79)	5.1 (1.5)	10.8 (3.9)
HbA1c	5.9 (0.52)	5.8 (0.57)	5.6 (0.26)	5.8 (0.32)
HOMA-IR	1.5 (0.29)	2.3 (0.98)	1.2 (0.37)	2.8 (1.14)

In the sub-sample (n = 21) with one-year follow-up measures, mean absolute muscle mass was 17.1 kg (± 2.7) and relative muscle mass was 26.1% (±5.2). Over the one-year follow-up, on average, muscle mass decreased 1.24% (± 5.8) and 1.0 kg (±3.7) in these 21 women. See Supplemental Appendix S3 in S1 File.

## Discussion

These results provide evidence for an association between D_3_Cr muscle mass and insulin-glucose homeostasis. Although prior research has examined this relationship using lean body mass from DXA, this is the first study using objectively measured D_3_Cr muscle mass in women. The data demonstrate that women with lower D_3_Cr muscle mass have higher values of circulating insulin, glucose, HbA1c levels along with HOMA-IR levels. We additionally report on sarcopenic obesity, defined by D_3_Cr muscle mass and body fat from DXA, in this population of older women. Sarcopenic obesity was prevalent in this population. The results provide preliminary support for muscle mass as a predictor of insulin-glucose homeostasis, independently of obesity status. Non-obese women with low muscle mass had higher blood glucose, insulin, HbA1c, and HOMA-IR values than non-obese women with high muscle mass. Admittedly, the magnitude of these differences were relatively small, but these findings support a hypotheses that can be tested in future research. Women who were obese had higher markers of insulin resistance, regardless of muscle mass. Finally, we also described sarcopenic obesity using D_3_Cr muscle mass and BMI >30kg/m^2^.

Prior research in older adults older adults (e.g., Health ABC [27], Baltimore Longitudinal Study of Aging [28], InCHIANTI [29]) has demonstrated T2D and impaired glucose tolerance are associated with an accelerated loss of lean body mass and poor physical function [30]. However, these studies used measures of lean body mass via DXA scan or bioelectrical impedance, not D_3_Cr measured muscle mass, and diagnosed T2D rather than serum analytes or biomarkers of subclinical disease [29, 31]. Our results demonstrate that women with low muscle mass had higher values of glucose, insulin, HbA1c, and HOMA-IR. We did not examine the relationship between muscle mass, insulin-glucose homeostasis, and functional outcomes (gait speed, strength, mobility-disability, frailty) but this is an important avenue for future research. The role of obesity in the relationship between muscle mass, T2D, and functional outcomes has not been explored. Orwoll and colleagues found that obesity had minimal effect on functional outcomes above and beyond the effects of D_3_Cr muscle mass, sarcopenic obesity was not associated with a greater risk of disability than sarcopenia alone [9].

There are several hypothesized pathophysiologic mechanisms connecting muscle mass, insulin resistance, and T2D [32]. Insulin resistance decreases muscle protein synthesis, resulting in an imbalance in the rate of muscle protein synthesis and muscle protein breakdown, affecting muscle size, quality, and function [32]. In the presence of insulin resistance, activation of the mTOR pathway is inhibited and autophagy is activated [16, 32, 33]. In rodent models, diabetes exacerbates age-related muscle loss, insulin-dependent activation pathways regulating protein synthesis are blunted in the muscle tissue of diabetic mice, and decreased muscle size may contribute to muscle atrophy before the onset of overt diabetes [34, 35].

There are two notable methodological challenges to address in research focused on muscle mass and diabetes: 1) the bidirectional relationship between muscle mass and diabetes and 2) disentangling the effects of aging and diabetes on loss of muscle mass. Insulin resistance drives autophagy, degradation of muscle protein, and mitochondrial dysfunction, all of which lead to loss of muscle mass and strength. Given the role of muscle mass in glucose uptake, loss of muscle mass has the potential to exacerbate insulin resistance, creating a feedback loop [13]. The bidirectional nature of this relationship requires specialized analytic techniques to properly assess the magnitude of hypothesized effects, such as marginal structural models to properly address time varying exposures and mediation analysis. The second consideration is the effect of aging on these relationships. Aging is the strongest risk factor for sarcopenia and the burden of diabetes is highest in older age groups. It is not yet clear how aging affects the relationship between muscle loss and diabetes, whether more muscle mass is lost among older adults with diabetes compared to age-matched non-diabetic peers, and the role of physical activity as a mediator or moderator of these relationships. The D_3_Cr dilution method has the potential to significantly advance our understanding of these topics in older adults, as accurate measurement of muscle mass is an essential pre-requisite to this research. There is an additional methodologic challenge for research on sarcopenic obesity, related to the need for a gold-standard definition of obesity for this construct [36, 37]. Our results highlight the variation in prevalence and associated measures of dysglycemia when different definitions are used.

This research used the D_3_Cr method to measure skeletal muscle mass remotely in a community-dwelling sample of postmenopausal women. There are several practical reasons why D_3_Cr muscle mass may be considered over DXA or other types of scan-based measures of body composition (e.g., CT, MRI), that require an in-person clinic visit. In this study of postmenopausal women, we found high acceptability (100% of participants were willing to take the D_3_Cr capsule) and demonstrated feasibility of two variations of the D_3_Cr dilution protocol. Initially, all women were provided the capsule at a study visit and were asked to return the urine sample via Fedex overnight shipping. In the one-year follow-up subgroup, the protocol was completely remote: women were mailed the D_3_Cr capsule to their homes and returned the urine sample via Fedex. D_3_Cr has been used previously to measure muscle mass in a large sample (n = 1376) of older men in the MrOS cohort [14]. However, the MrOS study did not include older women and the D_3_Cr method was implemented as part of a clinic visit [14]. Our results demonstrate that it is possible to obtain valid measures of muscle mass without requiring travel to a clinic setting for measurement. Travel and transportation are important barriers to study participation for older adults and may introduce selection bias [38]. In addition, using the D_3_Cr approach eliminates the need for expensive scanners (e.g., DXA, CT, or MRI) and trained technicians, and does not expose participants to radiation.

The results presented herein are from a pilot study, thus sample size was limited. A larger sample size will be required to thoroughly investigate the interaction between sarcopenia and obesity on diabetes, and potential connection to functional outcomes. The data collection period also spanned the initial phase of the COVID-19 pandemic: baseline data collection occurred in August 2019 and follow-up data collection occurred in August 2020. Stay-home public health orders in the Spring of 2020 may have impacted muscle mass in study participants. There are different plausible effects of these public health directives on muscle mass: some women may have been increasingly sedentary, relying on others for tasks such as grocery delivery, while others may have been more active, passing the time by doing chores or walking outdoors. In addition, the data on change in D_3_Cr muscle mass in the subset (n = 21) of participants should be interpreted cautiously given the small sample size. These data may be helpful to inform future hypotheses regarding the magnitude of 1-year change in D_3_Cr, but future research is needed in large, diverse samples of older adults.

Sarcopenia, obesity, and T2D are highly prevalent in older women and, given the high burden of T2D and the squelea of complications in aging populations, it is important to examine risk factors that can be modified via intervention. Skeletal muscle mass and obesity are both modifiable risk factors; research has demonstrated that diet and exercise interventions can improve muscle mass and decrease obesity levels [1]. Our results suggest some differences in physical activity levels among women with high and low muscle mass, but further examination, including more detailed measurement of physical activity levels in a larger sample of women is required. Strategies to preserve or enhance muscle mass may reduce the risk of T2D. Research is needed to examine the extent to which muscle mass and fat mass mediate the relationship between intervention strategies, the pathogenesis of impaired insulin-glucose homeostasis, and downstream consequences of T2D [30, 32, 39]. However, such research is not possible without valid measures of muscle mass. Obtaining measures of muscle mass in large-scale health research studies has long been a critical barrier to progress, but the D_3_Cr dilution approach has transformed our ability to measure muscle mass remotely. The use of D_3_Cr to measure muscle mass is an important advancement in clinical, epidemiologic, and population health research and has significant potential to contribute to healthy longevity in older adults.

## Supporting information

S1 File This is the supplementary appendix for the manuscript. (DOCX)
