# Supplementary material for: Association of muscle mass measured by D3-Creatine (D3Cr), sarcopenic obesity, and insulin-glucose homeostasis in postmenopausal women
Source: PLoS One. 2022 Dec 9;17(12):e0278723. doi: 10.1371/journal.pone.0278723 (PMC9733841; doi:10.1371/journal.pone.0278723)
Supplement: S1 File — (DOCX) [file pone.0278723.s001.docx]

**Supplementary Appendices**

**S1 Additional materials related to D3Cr remote collection procedure**

**A. Checklist for preparing study kit for participants**

1. Styrofoam mailer (in box) with freezer pack inside
2. Dipstick inside Styrofoam mailer

*PLEASE ENSURE DIPSTICK HAS PARTICIPANT ID LABEL ON IT*

1. Biohazard bag (with gloves inside)
2. Urine cup
3. Big white return shipping bag
4. Completed FedEx label

-- with date field highlighted and participant address completed

-- Before you put the FedEx label in their take-home kit, please photocopy address label so we have tracking number recorded in their chart

1. Paperwork

- Freezer reminder
- Instructions for providing sample
- Sample collection form

1. Once all of the items have been assembled and placed into a white WHI bag, please put a post-it sticky note on top of the package with the initials and visit start time of the participant. For example, if you assembled the kit for Jane Doe and her appointment is 8:15am, please write JD 08:15

**B. Freezer Reminder**


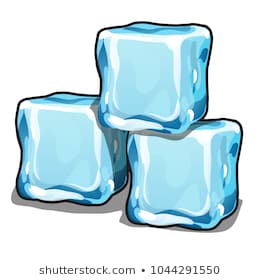
Please remember to put the cold pack (located inside the Styrofoam box) into your **freezer**.

C. **Sample Collection Form**

Please record the time and date you provided your urine sample:

Time: ____:______ AM

Date: ____/______/_______

We need this information for our records. Instructions on returning the urine to the clinic are on the next page.

**PLEASE MAKE SURE TO RETURN THIS PAPER WITH YOUR SPECIMEN**

S2: **Comparison of indices of insulin-glucose homeostasis (mean + SD ) and sarcopenic obesity defined by D_3_Cr muscle mass with (A) BMI and (B) Waist Circumference**

| (A) Sarcopenic obesity [with obesity defined by BMI >30 kg/m^2^)] | | | | |
| --- | --- | --- | --- | --- |
|  | Low muscle mass / non-obese  (n=26) | Low muscle mass / obese  (n=10) | High muscle mass / non-obese  (n=33) | High muscle mass / obese  (n=4) |
| Blood glucose | 104.7 (21.4) | 97.5 (8.5) | 99.7 (10.1) | 104.6 (11.2) |
| Insulin | 8.8 (4.0) | 6.7 (3.5) | 8.6 (3.2) | 11.7 (3.1) |
| HbA1c | 5.8 (0.65) | 5.7 (0.28) | 5.9 (0.31) | 6.0 (0.32) |
| HOMA-IR | 2.2 (1.0) | 1.6 (1.0) | 2.11 (0.37) | 3.0 (0.77) |
| (B) Sarcopenic obesity [with obesity defined by Waist Circumference >88cm)] | | | | |
|  | Low muscle mass / non-obese  (n=14) | Low muscle mass / obese  (n=22) | High muscle mass / non-obese  (n=27) | High muscle mass / obese  (n=10) |
| Blood glucose | 103.9 (12.9) | 95.7 (5.5) | 102.8 (20.9) | 106.3 (11.2) |
| Insulin | 7.1 (2.0) | 6.0 (2.6) | 9.4 (4.1) | 10.6 (5.0) |
| HbA1c | 5.9 (0.3) | 5.6 (0.2) | 5.8 (0.6) | 6.0 (0.3) |
| HOMA-IR | 1.8 (0.6) | 1.4 (0.7) | 2.4 (1.1) | 2.8 (1.4) |

**S3: Comparison of D_3_Cr Muscle mass at Time 1 (baseline) and Time 2 (1-year post baseline)
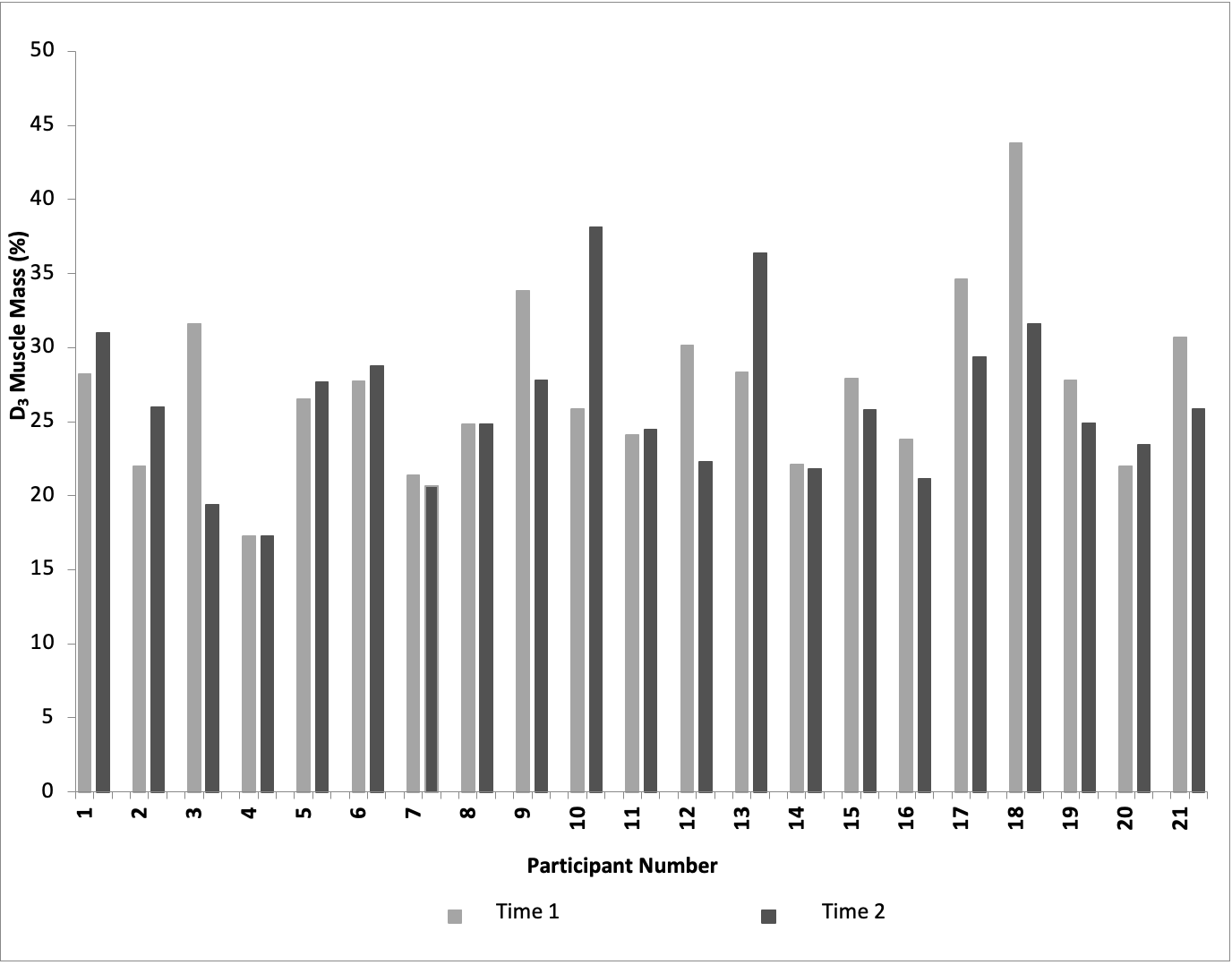
**

Time 2

Time 1
